# Author Correction: Asymmetry underlies stability in power grids

**DOI:** 10.1038/s41467-024-52495-z

**Published:** 2024-09-18

**Authors:** Ferenc Molnar, Takashi Nishikawa, Adilson E. Motter

**Affiliations:** 1 Department of Physics and Astronomy, Northwestern University, Evanston, IL USA; 2 Northwestern Institute on Complex Systems, Northwestern University, Evanston, IL USA; 3 Present Address: SimpleRose Inc, 1017 Olive Street, Suite 800, Saint Louis, MO 63101 USA

**Keywords:** Energy grids and networks, Complex networks, Nonlinear phenomena

Correction to: *Nature Communications* 10.1038/s41467-021-21290-5, published online 5 March 2021

In Fig. 1 of the original version of this Article, the values of *b*_1_, *b*_2_, and *b*_3_ for optimal damping were inadvertently rounded to a single decimal place, resulting in a parameter combination that does not approximate the stability of the global optima as intended. The actual parameter values used to generate the figure are *b*_1_ ≈ 2.47, *b*_2_ ≈ 3.17, *b*_3_ ≈ 1.47 (or equivalently, *b*_1_ ≈ 1.47, *b*_2_ ≈ 3.17, *b*_3_ ≈ 2.47). Rounding these values to two decimal places maintains the intended stability behavior. Figure 1 and its caption in both the PDF and HTML versions of the Article have now been updated with these approximations.

