# Supplementary material for: Asymmetry underlies stability in power grids
Source: Nat Commun. 2021 Mar 5;12:1457. doi: 10.1038/s41467-021-21290-5 (PMC7935983; doi:10.1038/s41467-021-21290-5)
Supplement: Supplementary file 1 — Supplementary Information [file 41467_2021_21290_MOESM1_ESM.pdf]

Supplementary Information

Asymmetry underlies stability in power grids

Supplementary Figures

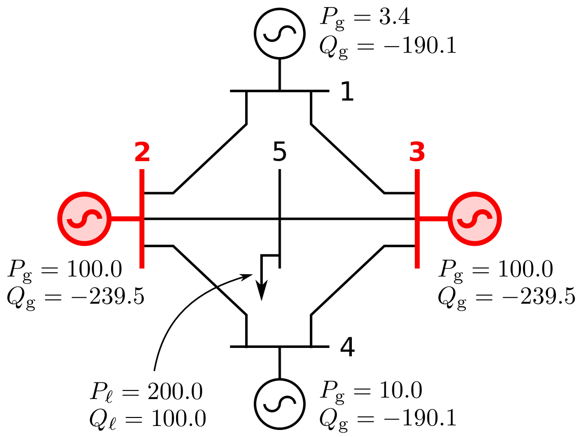

Supplementary Fig. 1: **System diagram for the 4-generator example system in Fig. 5.** The generators at nodes 1–4 produce active and reactive power P_g and Q_g (in MW and MVAR, respectively). The load at node 5 consumes active and reactive power P_ℓ and Q_ℓ. The other generator parameters are identical among nodes 1–4, except for the tunable damping parameters β_i, while the power line parameters are identical among all six lines connecting the nodes.

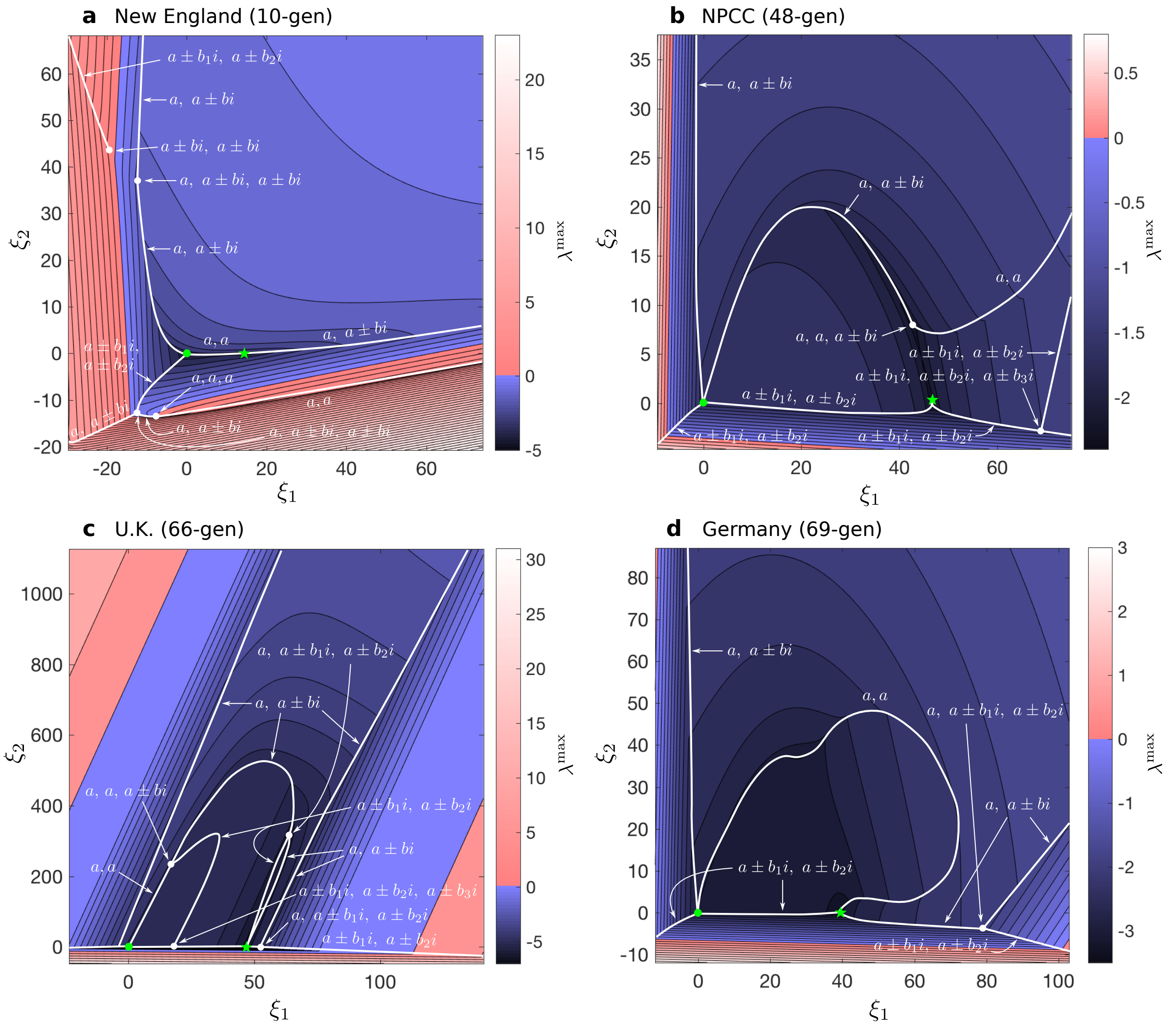

Supplementary Fig. 2: **Cusp hypersurfaces in the stability landscape of larger networks. a–d** Contour levels of $\lambda^{\max}$ on the plane M, defined as the hyperplane perpendicular to L that contains $\boldsymbol{\beta}_=$ and $\boldsymbol{\beta}_{\neq}$ (as in Fig. 2), for the four systems used in Fig. 6. The horizontal coordinate ξ_1 represents the Euclidean distance from the point $\boldsymbol{\beta}_=$ along the line connecting $\boldsymbol{\beta}_=$ to $\boldsymbol{\beta}_{\neq}$, and ξ_2 is the distance along the line orthogonal to the ξ_1-axis. A white curve indicates the (one-dimensional) cross section of a codimension-one cusp hypersurface and corresponds to single degeneracy of the real parts of the eigenvalues of the Jacobian $\mathbf{J}$, such as two identical real eigenvalues $\{a, a\}$, two pairs of complex conjugate eigenvalues with matching real parts $\{a \pm b_1 i,\ a \pm b_2 i\}$, and one real eigenvalue matching the real parts of a conjugate eigenvalue pair $\{a,\ a \pm bi\}$. The green dot and star indicate $\boldsymbol{\beta}_=$ and $\boldsymbol{\beta}_{\neq}$, respectively. These two points and the white dots correspond to the cross sections of cusp hypersurfaces of higher codimensions, each representing specific double or higher degeneracy of the real parts of the eigenvalues.

Supplementary Notes

Supplementary Note 1: Analysis of the stability landscape around $\boldsymbol{\beta}_=$

Our detailed analysis of the stability landscape around the point $\boldsymbol{\beta}_= = (\beta_=, \ldots, \beta_=)$, $\beta_= = 2\sqrt{\alpha_2}$, is divided into three parts. We first derive a formula for the maximum Lyapunov exponent $\lambda^{\max}$ in terms of the coefficients of the characteristic polynomial in a neighborhood of $\boldsymbol{\beta}_=$, which allows us to express a necessary condition for $\lambda^{\max}$ to decrease along a given path (Sec. 1). We then derive equations that relate these coefficients to $\boldsymbol{\beta}$ (Sec. 2). Finally, we apply the Implicit Function Theorem (IFT) to these equations and show that, for a generic system, any descending path must be tangent to a system-specific hyperplane in the $\boldsymbol{\beta}$-space (Sec. 3). In that section, we also derive an explicit formula for $\lambda^{\max}$ in terms of the parameterization of the path when the path is transverse to the hyperplane. Two example cases, $n = 2$ and $n = 3$, are presented for illustration in Sec. 4.

1 Maximum Lyapunov exponent $\boldsymbol{\lambda}^{\max}$ on arbitrary path through $\boldsymbol{\beta}_=$

In the main text, we introduced the $2n \times 2n$ Jacobian matrix $\mathbf{J}$ that characterizes the linearized dynamics of the generators. Recall that

$$\mathbf{J} = \begin{pmatrix} \mathbf{O} & \mathbf{I} \\ -\mathbf{P} & -\mathbf{B} \end{pmatrix}, \tag{1}$$

where $\mathbf{O}$ and $\mathbf{I}$ denote the null and the identity matrix of size n, respectively, the $n \times n$ matrix $\mathbf{P} = (P_{ik})$ is given by Eq. (3) in the main text, and $\mathbf{B}$ is the diagonal matrix of elements β_i. We define

$$\overline{\mathbf{J}} = \begin{pmatrix} \mathbf{O} & \mathbf{I} \\ -\mathbf{P}/\beta_=^2 & -\mathbf{B}/\beta_= \end{pmatrix}, \tag{2}$$

assuming $\beta_= > 0$ (or, equivalently, $\alpha_2 > 0$). Note that ν is an eigenvalue of $\overline{\mathbf{J}}$ if and only if $\beta_=\nu$ is an eigenvalue of $\mathbf{J}$, since the characteristic polynomial of $\overline{\mathbf{J}}$ can be written as

$$\begin{aligned} \det(\overline{\mathbf{J}} - \nu\mathbf{I}) &= \det(\nu^2\mathbf{I} + \nu\mathbf{B}/\beta_= + \mathbf{P}/\beta_=^2) \\ &= \beta_=^{-2n} \det(\beta_=^2\nu^2\mathbf{I} + \beta_=\nu\mathbf{B} + \mathbf{P}) \\ &= \beta_=^{-2n} \det(\mathbf{J} - \beta_=\nu\mathbf{I}). \end{aligned} \tag{3}$$

Consider a (possibly curved) path $\boldsymbol{\gamma}$ passing through the point $\boldsymbol{\beta}_=$ in the space of all $\boldsymbol{\beta}$, parametrized by ε through a differentiable vector function $\boldsymbol{\beta} = \boldsymbol{\gamma}(\varepsilon)$ satisfying $\boldsymbol{\gamma}(0) = \boldsymbol{\beta}_=$. We denote the parametrized eigenvalues of $\overline{\mathbf{J}}$ as $\nu_{j\pm} = \nu_{j\pm}(\varepsilon)$, $j = 1, \ldots, n$. The eigenvalues of $\mathbf{P}$ are denoted by $\alpha_1, \ldots, \alpha_n$, among which we have $\alpha_1 = 0$, and we assume that they are all real,

distinct, and indexed so that $0 < \alpha_2 < \cdots < \alpha_n$. The assumption that these eigenvalues are distinct will be crucial in the arguments that follow. When $\varepsilon = 0$, we have $\mathbf{B} = \beta_= \mathbf{I}$, so

$$\det(\overline{\mathbf{J}} - \nu \mathbf{I}) = \beta_=^{-2n} \det[\beta_=^2(\nu^2 + \nu)\mathbf{I} + \mathbf{P}], \tag{4}$$

and this implies that $\beta_=^2(\nu^2 + \nu) = \alpha_j$, or equivalently $\nu = \big(-1 \pm \sqrt{1 - \alpha_j/\alpha_2}\big)/2$, whenever ν is an eigenvalue of $\overline{\mathbf{J}}$. We thus index $\nu_{j_\pm}(\varepsilon)$ so that

$$\nu_{j_\pm}(\varepsilon) \xrightarrow{\varepsilon \to 0} \nu_{j_\pm}(0) = \frac{-1 \pm \sqrt{1 - \alpha_j/\alpha_2}}{2}, \quad j = 1, \ldots, n. \tag{5}$$

Note that $\nu_{1_+}(\varepsilon) \to \nu_{1_+}(0) = 0$ and $\nu_{1_-}(\varepsilon) \to \nu_{1_-}(0) = -1$ are not relevant for determining the stability of synchronous states since these eigenvalues are associated with perturbation modes that do not affect synchronization. Thus, the Lyapunov exponent $\lambda^{\max}$ determining the stability is the largest real component among the remaining eigenvalues:

$$\lambda^{\max}(\varepsilon) = \beta_= \cdot \max_{2 \le j \le n} \max\big\{Re(\nu_{j_+}(\varepsilon)), Re(\nu_{j_-}(\varepsilon))\big\}. \tag{6}$$

For $\varepsilon = 0$, which corresponds to the point $\boldsymbol{\beta}_=$, it follows from the formula for $\nu_{j_\pm}(0)$ in Eq. (5) and $\alpha_2 < \alpha_j$, $\forall j \ge 3$, that

$$\lambda^{\max}(0) = \beta_= \cdot Re(\nu_{2_+}(0)) = -\frac{\beta_=}{2} = -\sqrt{\alpha_2} = \lambda_=^{\max}. \tag{7}$$

Since eigenvalues are continuous functions of the matrix elements and $\boldsymbol{\gamma}$ is a continuous function, $\nu_{j_\pm}(\varepsilon)$ changes with ε continuously. Thus, for $\varepsilon \neq 0$, the eigenvalue $\nu_{2_+}(\varepsilon)$ determines the maximum in Eq. (6) for sufficiently small ε, and hence we have

$$\lambda^{\max}(\varepsilon) = \beta_= \cdot Re(\nu_{2_+}(\varepsilon)) = \beta_= \cdot Re\left(-\frac{c_2}{2} + \frac{1}{2}\sqrt{c_2^2 - 4d_2}\right), \tag{8}$$

where we factored the characteristic polynomial of $\overline{\mathbf{J}}$ into quadratic factors as

$$\det(\overline{\mathbf{J}} - \nu \mathbf{I}) = \big(\nu^2 + c_1\nu\big)\big(\nu^2 + c_2\nu + d_2\big) \cdots \big(\nu^2 + c_n\nu + d_n\big), \tag{9}$$

(noting that $d_1 = 0$ always holds because $\nu = 0$ is an eigenvalue of $\overline{J}$ associated with $\alpha_1 = 0$). Equations (8) and (9) imply that $\lambda^{\max}$ can be viewed as a function of c_2 and d_2 and thus defines a landscape over the (c_2, d_2)-plane, while the path $\boldsymbol{\gamma}(\varepsilon)$ in the $\boldsymbol{\beta}$-space corresponds a path on the (c_2, d_2)-plane defined by the functions $c_2 = c_2(\varepsilon)$ and $d_2 = d_2(\varepsilon)$. Defining $f(c_2, d_2) \equiv Re\big(-c_2 + \sqrt{c_2^2 - 4d_2}\big)/2$, we see that the condition for $\lambda^{\max}$ to be decreasing along the path $\boldsymbol{\gamma}(\varepsilon)$ starting at $\boldsymbol{\beta}_=$ in the $\boldsymbol{\beta}$-space is equivalent to the condition that the corresponding path $(c_2(\varepsilon), d_2(\varepsilon))$ starting at $(c_2, d_2) = (1, 1/4)$ immediately enters the following triangular region of the (c_2, d_2)-plane:

$$\big\{(c_2, d_2) :\ f(c_2, d_2) < -1/2\big\} = \big\{(c_2, d_2) :\ 2(d_2 - 1/4) > c_2 - 1 > 0\big\}. \tag{10}$$

The latter condition implies that the derivatives of $c_2(\varepsilon)$ and $d_2(\varepsilon)$ at $\varepsilon = 0$ satisfy $2d_2'(0) \geq c_2'(0)$. We may assume $c_2'(0) \geq 0$ without loss of generality. (If not, we simply need to make the change of variable, $\varepsilon \to -\varepsilon$.) In Sec. 3, we show that, for a generic choice of the matrix $\mathbf{P}$, we have $d_2'(0) = 0$. This implies that, if $\lambda^{\max}$ decreases along the path, we have $c_2'(0) = 0$. In general, if $c_2'(0) = d_2'(0) = 0$, the nonlinearity of $c_2(\varepsilon)$ and $d_2(\varepsilon)$ determines whether $\lambda^{\max}$ decreases along the path in either direction. We further show that the condition $c_2'(0) = 0$ translates to the path $\boldsymbol{\gamma}$ being tangent to a specific hyperplane, which we denote by L, at the point $\boldsymbol{\beta}_=$.

2 Equations relating coefficients c_i and d_i to parameter ε

We now derive equations that define the coefficients c_i and d_i implicitly as functions $c_i = c_i(\varepsilon)$ and $d_i = d_i(\varepsilon)$. We first rewrite the characteristic polynomial of $\overline{\mathbf{J}}$ as

$$\det(\overline{\mathbf{J}} - \nu\mathbf{I}) = \det(\nu^2\mathbf{I} + \nu\mathbf{B}/\beta_= + \mathbf{P}/\beta_=^2) = \det(\nu^2\mathbf{I} + \nu\overline{\mathbf{B}} + \mathbf{D}), \tag{11}$$

where we used a similarity transformation $\mathbf{Q}^{-1}(\mathbf{P}/\beta_=^2)\mathbf{Q} = \mathbf{D}$ and $\mathbf{B}/\beta_=$ as $\mathbf{Q}^{-1}(\mathbf{B}/\beta_=)\mathbf{Q} = \overline{\mathbf{B}}$ (based on the diagonalization of the matrix $\mathbf{P}$), and $\mathbf{D}$ is the diagonal matrix with diagonal elements $\overline{\alpha}_i \equiv \alpha_i/\beta_=^2 = \frac{1}{4}\alpha_i/\alpha_2$. Along the path $\boldsymbol{\gamma}(\varepsilon) = (\gamma_1(\varepsilon), \dots, \gamma_n(\varepsilon))$, the components of the matrix $\overline{\mathbf{B}}$ can be expressed as

$$\overline{B}_{ij}(\varepsilon) = \sum_{\ell=1}^{n} u_{i\ell} v_{j\ell} \gamma_\ell(\varepsilon)/\beta_=, \tag{12}$$

where $u_{i\ell}$ and $v_{i\ell}$ are the ℓth component of the left and right eigenvectors of $\mathbf{P}$ associated with the eigenvalue α_i, respectively. We now use the definition of the determinant,

$$\det(\mathbf{A}) \equiv \sum_\sigma \text{sign}(\sigma) \prod_{i=1}^{n} A_{i\sigma(i)} = \sum_\sigma \text{sign}(\sigma) \cdot A_{1\sigma(1)} \cdots A_{n\sigma(n)}, \tag{13}$$

where the summation $\sum_\sigma$ is taken over all possible permutations σ of indices, and sign(σ) is the sign of permutation σ (i.e., sign$(\sigma) = 1$ when σ is an even permutation and sign$(\sigma) = -1$ when σ is an odd permutation). With this definition, we can write the coefficient of the ν^k term (for $k = 1, \dots, 2n-1$; no constant term corresponding to $k = 0$, since $\nu = 0$ is an eigenvalue) of the polynomial in Eq. (11) as

$$\sum_\sigma \text{sign}(\sigma) \sum_{\{k_i\}} \prod_{i=1}^{n} E_{i\sigma(i)}^{(k_i)} \cdot \chi\big(\textstyle\sum_i k_i = k\big), \tag{14}$$

where the summation $\sum_{\{k_i\}}$ is taken over all possible combinations of $k_i = 0, 1, 2$, $i = 1, \dots, n$. The function χ is an indicator function defined by $\chi\big(\sum_i k_i = k\big) = 1$ if $\sum_i k_i = k$ and

$\chi\big(\sum_i k_i = k\big) = 0$ otherwise. The matrices $E_{ij}^{(k)}$ are defined for $k = 0, 1, 2$ as

$$E_{ij}^{(0)} = D_{ij} = \overline{\alpha}_i \delta_{ij}, \quad E_{ij}^{(1)} = \overline{B}_{ij}(\varepsilon), \quad E_{ij}^{(2)} = \delta_{ij}, \tag{15}$$

corresponding to the matrices $\mathbf{D}$, $\overline{\mathbf{B}}$, and $\mathbf{I}$ in Eq. (11), respectively (δ_{ij} is the Kronecker delta function). Equations (14) and (15) provide an expression for the coefficients of $\det(\overline{\mathbf{J}} - \nu\mathbf{I})$ in terms of $\boldsymbol{\beta}$, and thus of ε, for a given system and its matrix $\mathbf{P}$.

We now derive a different expression for $\det(\overline{\mathbf{J}} - \nu\mathbf{I})$, this time in terms of the characteristic polynomial coefficients c_i and d_i, using Eq. (9). Ignoring the dependence of c_i and d_i on ε for the moment and regarding them as independent variables, the coefficient of the ν^k term can be written as

$$\sum_{\{k_i\}} \prod_{i=1}^{n} a_i^{(k_i)} \cdot \chi\big(\textstyle\sum_i k_i = k\big), \tag{16}$$

where the summation is the same as in Eq. (14) and

$$a_i^{(0)} = d_i, \quad a_i^{(1)} = c_i, \quad a_i^{(2)} = 1. \tag{17}$$

Setting Eqs. (14) and (16) equal to each other, we obtain a set of nonlinear equations that must be satisfied by the variables $c_1, \ldots, c_n$, $d_1, \ldots, d_n$, and ε:

$$F_k(c_1, \ldots, c_n, d_1, \ldots, d_n, \varepsilon) = 0, \quad k = 1, 2, \ldots, 2n-1, \tag{18}$$

or, in vector form,

$$\mathbf{F}(c_1, \ldots, c_n, d_1, \ldots, d_n, \varepsilon) = \mathbf{0}, \tag{19}$$

where we have defined $\mathbf{F} \equiv (F_1, \ldots, F_{2n-1})^T$, and the functions F_k are given by

$$\begin{aligned} F_k &= \sum_{\{k_i\}} \prod_{i=1}^{n} a_i^{(k_i)} \cdot \chi\big(\textstyle\sum_i k_i = k\big) - \sum_{\sigma} \mathrm{sign}(\sigma) \sum_{\{k_i\}} \prod_{i=1}^{n} E_{i\sigma(i)}^{(k_i)} \cdot \chi\big(\textstyle\sum_i k_i = k\big) \\ &= \sum_{\{k_i\}} \chi\big(\textstyle\sum_i k_i = k\big) \cdot \left[\prod_{i=1}^{n} a_i^{(k_i)} - \sum_{\sigma} \mathrm{sign}(\sigma) \prod_{i=1}^{n} E_{i\sigma(i)}^{(k_i)} \right]. \end{aligned} \tag{20}$$

This is the equation that implicitly defines the functions $c_i = c_i(\varepsilon)$ and $d_i = d_i(\varepsilon)$. Note that, when $c_i = 1$, $d_i = \overline{\alpha}_i$, and $\varepsilon = 0$ (corresponding to the point $\boldsymbol{\beta}_=$), we have $E_{ij}^{(1)} = \overline{B}_{ij}(0) = \delta_{ij}$, and hence

$$a_i^{(0)} = d_i(0) = \overline{\alpha}_i, \quad a_i^{(1)} = c_i(0) = 1, \quad a_i^{(2)} = 1, \quad E_{ij}^{(k)} = a_i^{(k)} \delta_{ij}, \tag{21}$$

implying

$$\begin{aligned} F_k(1, \ldots, 1, \overline{\alpha}_1, \ldots, \overline{\alpha}_n, 0) &= \sum_{\{k_i\}} \chi\big(\textstyle\sum_i k_i = k\big) \cdot \left[\prod_{i=1}^{n} a_i^{(k_i)} - \sum_{\sigma} \mathrm{sign}(\sigma) \prod_{i=1}^{n} a_{i\sigma(i)}^{(k_i)} \delta_{i\sigma(i)} \right] \\ &= \sum_{\{k_i\}} \chi\big(\textstyle\sum_i k_i = k\big) \cdot \left[\prod_{i=1}^{n} a_i^{(k_i)} - \prod_{i=1}^{n} a_i^{(k_i)} \right] = 0, \end{aligned}$$

i.e., Eq. (18) is satisfied. The functions $c_i(\varepsilon)$ and $d_i(\varepsilon)$ defined through Eq. (20) thus satisfy $c_i(0) = 1$ and $d_i(0) = \overline{\alpha}_i$.

3 Characterizing descending paths on $\lambda^{\max}$-landscape

Here, we will apply the IFT to show that the functions $c_i(\varepsilon)$ and $d_i(\varepsilon)$ are continuously differentiable for small ε (and thus define a smooth curve in the neighborhood of the point $(1, \overline{\alpha}_i)$ in the (c_i, d_i)-plane), and we determine their first derivatives. The condition under which we can apply the IFT to Eq. (18) at the point $(c_1, \ldots, c_n, d_1, \ldots, d_n, \varepsilon) = (1, \ldots, 1, \overline{\alpha}_1, \ldots, \overline{\alpha}_n, 0)$ is that the $(2n-1) \times (2n-1)$ matrix

$$\mathbf{G} \equiv \begin{pmatrix} \frac{\partial F_1}{\partial c_1} & \cdots & \frac{\partial F_1}{\partial c_n} & \frac{\partial F_1}{\partial d_2} & \cdots & \frac{\partial F_1}{\partial d_n} \\ \vdots & & \vdots & \vdots & & \vdots \\ \frac{\partial F_{2n-1}}{\partial c_1} & \cdots & \frac{\partial F_{2n-1}}{\partial c_n} & \frac{\partial F_{2n-1}}{\partial d_2} & \cdots & \frac{\partial F_{2n-1}}{\partial d_n} \end{pmatrix}, \tag{22}$$

is non-singular, where the elements of $\mathbf{G}$ are all evaluated at that point. We note that d_1 is excluded from the set of variables here because $d_1 = 0$ always holds. We also note that $\mathbf{G}$ (and whether it is singular or not) is completely determined by $\overline{\alpha}_2, \ldots, \overline{\alpha}_n$, and hence by the matrix $\mathbf{P}$ (see examples in Sec. 4). For notational convenience, define $x_s = c_s$ for $s = 1, \ldots, n$ and $x_s = d_{s-n+1}$ for $s = n+1, \ldots, 2n-1$. Differentiating Eq. (20), we find an expression for the (k, s)-element of $\mathbf{G}$:

$$\begin{aligned} G_{ks} &= \frac{\partial F_k}{\partial x_s}(1, \ldots, 1, \overline{\alpha}_1, \ldots, \overline{\alpha}_n, 0) \\ &= \begin{cases} \displaystyle\sum_{\{k_i\}} \prod_{i \neq s} a_i^{(k_i)} \cdot \chi\big(s, k, \{k_i\}\big), & \text{if } s = 1, \ldots, n, \\ \displaystyle\sum_{\{k_i\}} \prod_{i \neq \hat{s}} a_i^{(k_i)} \cdot \chi\big(s, k, \{k_i\}\big), & \text{if } s = n+1, \ldots, 2n-1, \end{cases} \end{aligned} \tag{23}$$

where the summation is defined as in Eq. (14); we denote $\hat{s} \equiv s - n + 1$; the values of $a_i^{(k_i)}$ are given by Eq. (21); and we have defined

$$\chi\big(s, k, \{k_i\}\big) \equiv \begin{cases} 1 & \text{if } \sum_i k_i = k,\ k_s = 1, \text{ and } s = 1, \ldots, n, \\ 1 & \text{if } \sum_i k_i = k,\ k_{\hat{s}} = 0, \text{ and } s = n+1, \ldots, 2n-1, \\ 0 & \text{otherwise.} \end{cases} \tag{24}$$

Since the eigenvalues of $\mathbf{P}$ are assumed to be distinct, we have $\frac{1}{4} = \overline{\alpha}_2 < \cdots < \overline{\alpha}_n$. We have numerically verified that this condition holds for all networks considered in the main text. We first seek to show that the matrix $\mathbf{G}$ is non-singular by proving that the sth and s'th columns

of $\mathbf{G}$ are linearly independent for all distinct pairs s and s'. To do this, we first note that Eq. (23) simplifies in the special cases $k = 1$, $k = 2$, $k = 2n - 2$, and $k = 2n - 1$, as follows.

For $k = 1$, to satisfy $\sum_i k_i = k$, we must have $k_i = 1$ for exactly one value of i and $k_i = 0$ for all the others (recall that each k_i is either 0, 1, or 2). For the case $1 \leq s \leq n$, we can derive a simplified formula, but it is not needed below, so we will skip that case here. For the case $n+1 \leq s \leq 2n-1$, to have $\chi\big(s, k, \{k_i\}\big) = 1$ in Eq. (24) we must have $k_t = 1$ for some $t \neq \hat{s}$ and $k_i = 0$ for all $i \neq t$ (including $i = \hat{s}$). Since there are $n - 1$ possibilities for t, there are that many nonzero terms in the summation in Eq. (23), which reduces to

$$G_{1s} = \sum_{t \neq \hat{s}} \prod_{i \neq \hat{s}} a_i^{(k_i)} = \sum_{t \neq \hat{s}} \prod_{i \neq t, \hat{s}} \overline{\alpha}_i, \quad n+1 \leq s \leq 2n-1. \tag{25}$$

For $k = 2$, the condition $\sum_i k_i = k$ implies that we either have (a) $k_i = 2$ for exactly one value of i and $k_i = 0$ for all the others, or (b) $k_i = 1$ for two different values of i, and $k_i = 0$ for all the others. For $1 \leq s \leq n$, we have $\chi\big(s, k, \{k_i\}\big) = 0$ for the terms in Eq. (23) corresponding to case (a), according to Eq. (24). For nonzero terms in Eq. (23) corresponding to case (b), we have $k_s = 1$, $k_t = 1$ with some $t \neq s$, and $k_i = 0$ for all $i \neq s, t$. Putting the two cases together, we obtain

$$G_{2s} = \sum_{t \neq s} \prod_{i \neq s} a_i^{(k_i)} = \sum_{t \neq s} \prod_{i \neq t, s} \overline{\alpha}_i, \quad 1 \leq s \leq n. \tag{26}$$

A simplified expression for G_{2s} for $n+1 \leq s \leq 2n-1$ can also be obtained, but it is not needed for our purpose.

For $k = 2n - 2$, satisfying $\sum_i k_i = k$ requires that either (a) $k_i = 0$ for exactly one value of i and $k_i = 2$ for all the others, or (b) $k_i = 1$ for two different values of i, and $k_i = 2$ for all the others. For $1 \leq s \leq n$, similarly to the case of $k = 2$, there is no nonzero term in Eq. (23) corresponding to case (a), and for the nonzero terms in Eq. (23) corresponding to case (b), we have $k_s = 1$, $k_t = 1$ with some $t \neq s$, and $k_i = 2$ for all $i \neq s, t$. Putting these two cases together, we obtain

$$G_{2n-2,s} = \sum_{t \neq s} \prod_{i \neq s} a_i^{(k_i)} = \sum_{t \neq s} 1 = n - 1, \quad 1 \leq s \leq n. \tag{27}$$

For $n+1 \leq s \leq 2n-1$, case (b) does not correspond to any nonzero term in Eq. (23) because $k_i \neq 0$ for all i. Case (a), on the other hand, yields exactly one term with $k_{\hat{s}} = 0$, leading to

$$G_{2n-2,s} = \prod_{i \neq \hat{s}} a_i^{(k_i)} = 1, \quad n+1 \leq s \leq 2n-1. \tag{28}$$

For $k = 2n-1$, the only way to satisfy $\sum_i k_i = k$ is to have just one $k_i = 1$ and all the other $k_i = 2$. For $1 \leq s \leq n$, we must have $k_s = 1$ for a nonzero term, so only one term survives in Eq. (23), and hence

$$G_{2n-1,s} = \prod_{i \neq s} a_i^{(k_i)} = 1, \quad 1 \leq s \leq n. \tag{29}$$

For $n+1 \leq s \leq 2n-1$, there is no nonzero term since there is no i for which $k_i = 0$ (see Eq. (24)), and thus

$$G_{2n-1,s} = 0, \quad n+1 \leq s \leq 2n-1. \tag{30}$$

Now we can use Eqs. (25)–(30) to show that any pair of columns of $\mathbf{G}$ are linearly independent. For $1 \leq s \leq n$ and $n+1 \leq s' \leq 2n-1$, the last two components ($k = 2n-2$ and $k = 2n-1$) of the sth and s'th column vectors form the two-dimensional vectors $(n-1,1)^T$ and $(1,0)^T$, respectively, which are linearly independent. This implies that the full $(2n-1)$-dimensional vectors in the sth and s'th columns of $\mathbf{G}$ are also linearly independent. For $1 \leq s < s' \leq n$, since the last components ($k = 2n-1$) of the sth and s'th column vectors are both equal to one, it suffices to show that the second component ($k = 2$) is different in order to establish that they are linearly independent. From Eq. (26), we have

$$\begin{aligned} G_{2s} - G_{2s'} &= \sum_{t \neq s} \prod_{i \neq t,s} \overline{\alpha}_i - \sum_{t \neq s'} \prod_{i \neq t,s'} \overline{\alpha}_i \\ &= \Big(\sum_{t \neq s,s'} \prod_{i \neq t,s} \overline{\alpha}_i + \prod_{i \neq s',s} \overline{\alpha}_i \Big) - \Big(\sum_{t \neq s,s'} \prod_{i \neq t,s'} \overline{\alpha}_i + \prod_{i \neq s,s'} \overline{\alpha}_i \Big) \\ &= \sum_{t \neq s,s'} \Big(\prod_{i \neq t,s,s'} \overline{\alpha}_i \Big) (\overline{\alpha}_{s'} - \overline{\alpha}_s) > 0, \end{aligned} \tag{31}$$

since $\overline{\alpha}_{s'} > \overline{\alpha}_s$ and $\overline{\alpha}_i > 0$, $\forall i$, and hence all terms in the summation are positive. Thus, we have $G_{2s} \neq G_{2s'}$, implying that the sth and s'th column vectors are linearly independent. For $n+1 \leq s < s' \leq 2n-1$, the argument is similar to the case of $1 \leq s < s' \leq n$; it suffices to show that the first component ($k = 1$) is different, and Eq. (25) yields

$$\begin{aligned} G_{1s} - G_{1s'} &= \sum_{t \neq \hat{s}} \prod_{i \neq t,\hat{s}} \overline{\alpha}_i - \sum_{t \neq \hat{s}'} \prod_{i \neq t,\hat{s}'} \overline{\alpha}_i \\ &= \sum_{t \neq \hat{s},\hat{s}'} \Big(\prod_{i \neq t,\hat{s},\hat{s}'} \overline{\alpha}_i \Big) (\overline{\alpha}_{\hat{s}'} - \overline{\alpha}_{\hat{s}}) > 0, \end{aligned} \tag{32}$$

where we have defined $\hat{s}' \equiv s' - n + 1$ (and recall that $\hat{s} = s - n + 1$). Combining all of the above, we have that the sth and s'th column vectors of $\mathbf{G}$ are linearly independent for all pairs of distinct s and s', which establishes that $\mathbf{G}$ is non-singular.

The IFT can now be applied to Eq. (18) to conclude that the functions $c_i(\varepsilon)$ and $d_i(\varepsilon)$ are continuously differentiable. Furthermore, their derivatives satisfy the set of equations obtained by substituting these functions into Eq. (18) and differentiating both sides with respect to ε:

$$\frac{d}{d\varepsilon} F_k(c_1(\varepsilon), \ldots, c_n(\varepsilon), d_1(\varepsilon), \ldots, d_n(\varepsilon), \varepsilon) = 0. \tag{33}$$

Using Eq. (20), this can be written as

$$\sum_{\{k_i\}} \chi\left(\sum_i k_i = k\right) \cdot \sum_{\ell=1}^{n} \left[\left(\prod_{i \neq \ell} a_i^{(k_i)}(\varepsilon) \right) \cdot \frac{d a_\ell^{(k_\ell)}(\varepsilon)}{d\varepsilon} - \right.$$
$$\left. - \sum_\sigma \text{sign}(\sigma) \left(\prod_{i \neq \ell} E_{i\sigma(i)}^{(k_i)}(\varepsilon) \right) \cdot \frac{d E_{\ell\sigma(\ell)}^{(k_\ell)}(\varepsilon)}{d\varepsilon} \right] = 0, \quad (34)$$

where we have now written the dependence of $a_i^{(k_i)}$ on ε explicitly. Setting $\varepsilon = 0$ and using Eq. (21), we obtain

$$\sum_{\ell=1}^{n} \left(\sum_{\{k_i\}} \prod_{i \neq \ell} a_i^{(k_i)}(0) \cdot \chi\left(\sum_i k_i = k\right) \right) \cdot \left[\frac{d a_\ell^{(k_\ell)}(0)}{d\varepsilon} - \frac{d E_{\ell\ell}^{(k_\ell)}(0)}{d\varepsilon} \right] = 0. \quad (35)$$

Noting that $k_\ell = 0, 1, 2$ and that the derivatives are both zero if $k_\ell = 2$ (see Eqs. (15) and (17)), we can rearrange the summation in Eq. (35) to write

$$\sum_{s=1}^{n} \left(\sum_{\{k_i\}} \prod_{i \neq s} a_i^{(k_i)}(0) \cdot \chi\big(s, k, \{k_i\}\big) \right) \cdot [x_s'(0) - y_s'(0)]$$
$$+ \sum_{s=n+1}^{2n-1} \left(\sum_{\{k_i\}} \prod_{i \neq \hat{s}} a_i^{(k_i)}(0) \cdot \chi\big(s, k, \{k_i\}\big) \right) \cdot [x_s'(0) - y_s'(0)] = 0, \quad (36)$$

where we recall that $x_s(\varepsilon) = c_s(\varepsilon) = a_s^{(1)}(\varepsilon)$ for $s = 1, \ldots, n$ and $x_s(\varepsilon) = d_{s-n+1}(\varepsilon) = a_s^{(0)}(\varepsilon)$ for $s = n+1, \ldots, 2n-1$, and we use the notations $y_s(\varepsilon) = \overline{B}_{ss}(\varepsilon)$ for $s = 1, \ldots, n$ and $y_s(\varepsilon) = \overline{\alpha}_{s-n+1}$ for $s = n+1, \ldots, 2n-1$. From Eq. (23), we see that Eq. (36) is equivalent to

$$\sum_{s=1}^{2n-1} G_{ks}[x_s'(0) - y_s'(0)] = 0, \quad (37)$$

which can be put in vector form as $\mathbf{G}(\mathbf{x}-\mathbf{y}) = \mathbf{0}$ using the notations $\mathbf{x} = (x_1'(0), \ldots, x_{2n-1}'(0))^T$ and $\mathbf{y} = (y_1'(0), \ldots, y_{2n-1}'(0))^T$. Thus, since $\mathbf{G}$ is non-singular, we have $\mathbf{x} = \mathbf{y}$, and hence $x_i'(0) = y_i'(0)$. It then follows that

$$c_i'(0) = x_i'(0) = y_i'(0) = \frac{d\overline{B}_{ii}(0)}{d\varepsilon} = \frac{d}{d\varepsilon} \left(\sum_{\ell=1}^{n} u_{i\ell} v_{i\ell} \gamma_\ell(\varepsilon) / \beta_= \right) \Bigg|_{\varepsilon=0} = \sum_{\ell=1}^{n} u_{i\ell} v_{i\ell} \gamma_\ell'(0) / \beta_= \quad (38)$$

for $i = 1, \ldots, n$, where we used the expression for $\overline{B}_{ij}$ from Eq. (12). It also follows that

$$d_i'(0) = x_{i+n-1}'(0) = y_{i+n-1}'(0) = \frac{d}{d\varepsilon} (\overline{\alpha}_i) \Bigg|_{\varepsilon=0} = 0 \quad (39)$$

for $i = 1, \ldots, n$, since $\overline{\alpha}_i$ is a constant that does not depend on ε. In particular, we have

$$c_2'(0) = \sum_{\ell=1}^{n} u_{2\ell} v_{2\ell} \gamma_\ell'(0)/\beta_= \quad \text{and} \quad d_2'(0) = 0. \tag{40}$$

Now, recall the argument at the end of Sec. 1 that $d_2'(0) = 0$ implies $c_2'(0) = 0$ if $\lambda^{\max}$ decreases along the path $\boldsymbol{\gamma}(\varepsilon)$ in the $\boldsymbol{\beta}$-space. In view of that argument, we see from Eq. (40) that, if the eigenvalues of $\mathbf{P}$ are all distinct, the vector $(\gamma_1'(0), \ldots, \gamma_n'(0))^T$ is parallel to the hyperplane L whenever $\lambda^{\max}$ decreases along the path, where L is uniquely defined by the equation

$$\sum_{i=1}^{n} u_{2i} v_{2i} (\beta_i - \beta_=) = 0. \tag{41}$$

In other words, any descending path on the $\lambda^{\max}$-landscape must be tangent to the hyperplane L at $\boldsymbol{\beta}_=$.

If the path $\boldsymbol{\gamma}(\varepsilon)$ is not tangent to L (and hence $c_2'(0) \neq 0$), the expansion of $c_2(\varepsilon)$ and $d_2(\varepsilon)$ around $\varepsilon = 0$, which reads

$$\begin{aligned} c_2(\varepsilon) &= 1 + c_2'(0)\varepsilon + O(\varepsilon^2), \\ d_2(\varepsilon) &= \frac{1}{4} + O(\varepsilon^2), \end{aligned} \tag{42}$$

can be substituted into Eq. (8) to obtain the following approximation for $\lambda^{\max}(\varepsilon)$:

$$\lambda^{\max}(\varepsilon) = \begin{cases} \lambda_=^{\max} - \beta_= c_2'(0)\varepsilon/2 + O(\varepsilon^2), & \varepsilon \le 0, \\ \lambda_=^{\max} + \beta_= \sqrt{c_2'(0)\varepsilon/2} + O(\varepsilon), & \varepsilon > 0. \end{cases} \tag{43}$$

(Recall we are assuming $c_2'(0) \geq 0$.) This establishes that the point $\boldsymbol{\beta}_=$ is a local minimizer of $\lambda^{\max}$ along any path that transversally intersects with the hyperplane L at $\boldsymbol{\beta}_=$.

4 Explicit calculation of matrix G for small n

Here, we derive an expression for the matrix $\mathbf{G}$ in Eq. (22) for $n = 2$ and for $n = 3$. As in Sec. 1–3 above, we assume $\beta_= > 0$, or equivalently, $\alpha_2 > 0$.

Example 1: For $n = 2$, Eq. (11) becomes

$$\begin{aligned} \det(\overline{\mathbf{J}} - \nu\mathbf{I}) = \det(\nu^2\mathbf{I} + \nu\overline{\mathbf{B}} + \mathbf{D}) &= \det \begin{pmatrix} \nu^2 + \overline{B}_{11}\nu & \overline{B}_{12}\nu \\ \overline{B}_{21}\nu & \nu^2 + \overline{B}_{22}\nu + 1/4 \end{pmatrix} \\ &= (\nu^2 + \overline{B}_{11}\nu)(\nu^2 + \overline{B}_{22}\nu + 1/4) - \overline{B}_{12}\overline{B}_{21}\nu^2 \\ &= \nu^4 + (\overline{B}_{11} + \overline{B}_{22})\nu^3 + (\overline{B}_{11}\overline{B}_{22} - \overline{B}_{12}\overline{B}_{21} + 1/4)\nu^2 + \overline{B}_{11}\nu/4, \\ &= \nu^4 + \frac{\beta_1 + \beta_2}{\beta_=}\nu^3 + \left(\frac{\beta_1\beta_2}{\beta_=^2} + \frac{1}{4}\right)\nu^2 + \overline{B}_{11}\nu/4, \end{aligned}$$

while Eq. (9) becomes

$$\begin{aligned}\det(\overline{\mathbf{J}} - \nu\mathbf{I}) &= (\nu^2 + c_1\nu)(\nu^2 + c_2\nu + d_2) \\ &= \nu^4 + (c_1 + c_2)\nu^3 + (c_1c_2 + d_2)\nu^2 + c_1d_2\nu.\end{aligned} \tag{44}$$

Thus, by comparing coefficients for the same powers of ν, we have

$$\begin{aligned}F_1(c_1, c_2, d_2, \varepsilon) &= c_1d_2 - \overline{B}_{11}/4, \\ F_2(c_1, c_2, d_2, \varepsilon) &= c_1c_2 + d_2 - \left(\frac{\beta_1\beta_2}{\beta_=^2} + \frac{1}{4}\right), \\ F_3(c_1, c_2, d_2, \varepsilon) &= c_1 + c_2 - \frac{\beta_1 + \beta_2}{\beta_=},\end{aligned}$$

and

$$\begin{aligned}\frac{\partial F_1}{\partial c_1} &= d_2, & \frac{\partial F_1}{\partial c_2} &= 0, & \frac{\partial F_1}{\partial d_2} &= c_1, \\ \frac{\partial F_2}{\partial c_1} &= c_2, & \frac{\partial F_2}{\partial c_2} &= c_1, & \frac{\partial F_2}{\partial d_2} &= 1, \\ \frac{\partial F_3}{\partial c_1} &= 1, & \frac{\partial F_3}{\partial c_2} &= 1, & \frac{\partial F_3}{\partial d_2} &= 0,\end{aligned}$$

$$\frac{\partial \mathbf{F}}{\partial(c, d)} = \begin{pmatrix} d_2 & 0 & c_1 \\ c_2 & c_1 & 1 \\ 1 & 1 & 0 \end{pmatrix}, \quad \mathbf{G} = \left.\frac{\partial \mathbf{F}}{\partial(c, d)}\right|_{\varepsilon=0} = \begin{pmatrix} 1/4 & 0 & 1 \\ 1 & 1 & 1 \\ 1 & 1 & 0 \end{pmatrix}.$$

Therefore, $\mathbf{G}$ is non-singular (regardless of the value of $\alpha_2 > 0$).

Example 2: For $n = 3$, it is convenient to first write out the terms in the first sum of the first line in Eq. (20) and differentiate them with respect to c_i and d_i (since the second sum does not contain c_i or d_i). The terms from the first sum are

$$F_1 = c_1d_2d_3 - \cdots, \tag{45}$$

$$F_2 = d_2d_3 + c_1c_2d_3 + c_1d_2c_3 - \cdots, \tag{46}$$

$$F_3 = c_1c_2c_3 + c_1d_3 + c_1d_2 + c_2d_3 + d_2c_3 - \cdots, \tag{47}$$

$$F_4 = d_2 + d_3 + c_1c_2 + c_2c_3 + c_3c_1 - \cdots, \tag{48}$$

$$F_5 = c_1 + c_2 + c_3 - \cdots. \tag{49}$$

Using this, we see that the matrix of partial derivatives is

$$\frac{\partial \mathbf{F}}{\partial(c, d)} = \begin{pmatrix} d_2d_3 & 0 & 0 & c_1d_3 & c_1d_2 \\ c_2d_3 + d_2c_3 & c_1d_3 & c_1d_2 & d_3 + c_1c_3 & d_2 + c_1c_2 \\ c_2c_3 + d_2 + d_3 & c_1c_3 + d_3 & c_1c_2 + d_2 & c_1 + c_3 & c_1 + c_2 \\ c_2 + c_3 & c_3 + c_1 & c_1 + c_2 & 1 & 1 \\ 1 & 1 & 1 & 0 & 0 \end{pmatrix}, \tag{50}$$

from which we obtain the matrix $\mathbf{G}$ by setting $c_1 = c_2 = c_3 = 1$, $d_2 = \overline{\alpha}_2 = 1/4$, and $d_3 = \overline{\alpha}_3$:

$$\mathbf{G} = \begin{pmatrix} \overline{\alpha}_3/4 & 0 & 0 & \overline{\alpha}_3 & 1/4 \\ 1/4 + \overline{\alpha}_3 & \overline{\alpha}_3 & 1/4 & 1 + \overline{\alpha}_3 & 5/4 \\ 5/4 + \overline{\alpha}_3 & 1 + \overline{\alpha}_3 & 5/4 & 2 & 2 \\ 2 & 2 & 2 & 1 & 1 \\ 1 & 1 & 1 & 0 & 0 \end{pmatrix}. \tag{51}$$

From this, we obtain $\det(\mathbf{G}) = -\overline{\alpha}_3(1 - 4\overline{\alpha}_3)^2/64 = -\alpha_3(\alpha_2 - \alpha_3)^2/(256\alpha_2^3)$ and see that $\mathbf{G}$ is non-singular if and only if $0 < \alpha_2 < \alpha_3$. This condition is indeed satisfied by the 3-generator system considered in the main text ($0 < \alpha_2 \approx 75.5 < \alpha_3 \approx 178.5$).
